# Does orthodontic postgraduate education influence the choice of orthodontic treatment?

**DOI:** 10.3389/fdmed.2025.1665422

**Published:** 2025-10-07

**Authors:** Sabina Saccomanno, Stefano Saran, Maria Teresa Petricca, Elena Caramaschi, Laura Ferrante, Francesco Inchingolo, Angelo Michele Inchingolo, Andrea Palermo, Gianna Dipalma, Alessio Danilo Inchingolo

**Affiliations:** ^1^ Department of Life Science, Health and Health Professions Link Italy, Rome, Italy; ^2^ Department of Human Sciences, Innovation and Territory, School of Dentistry, Postgraduate of orthodontics, University of Insubria, Varese, Italy; ^3^ Department of Medical, Oral and Biotechnological Sciences, G. D’Annunzio University of Chieti -Pescara, Chieti, Italy; ^4^ Private Practice, Parma, Italy; ^5^ Interdisciplinary Department of Medicine, University of Bari “Aldo Moro”, Bari, Italy; ^6^ Department of Biomedical, Surgical and Dental Sciences, Milan University, Milan, Italy; ^7^ Department of Experimental Medicine, University of Salento, Lecce, Italy

**Keywords:** orthodontics, post-graduate education, malocclusion, invisible aligners, questionnaire, functional therapy

## Abstract

**Aim:**

The purpose of this study is to understand how the training of a clinician influences his or her therapeutic choices in the orthodontic field.

**Materials and methods:**

An anonymized questionnaire was submitted to 317 Italian dentists to ask them about their training and what orthodontic therapies they perform. The answers were processed by statistical analysis.

**Results:**

221 of 314 respondents (70.3%) had an orthodontic postgraduate education and 93 subjects did not (29.7%). Out of the whole sample, 242 clinicians use functional therapy (i.e., Frankel, Bionator or Andresen), but while 133 of them, after functional therapy, apply both fixed orthodontic appliances (i.e., Straight wire, Tweed or Rickets) and aligners, 79 use only fixed oral appliances, and 19 dentists use only an aligner. The application of a lingual technique is perfectly independent from having an orthodontic postgraduate education or not.

**Conclusion:**

Differences were found between dentists with an orthodontic postgraduate education and dentists without it. Most dentists in Italy pursued a postgraduate education. In addition, most orthodontists are dedicated exclusively to orthodontics in their office, while dentists who don't have an orthodontic postgraduate education do not practice orthodontics exclusively in their offices. It is possible to conclude that pursuing a specialization in orthodontics determines advantages for both practitioners and patients: it gives orthodontists those extra skills to customize a diagnosis and daily treatments in a more precise and innovative way, using a wider variety of therapeutic options and relying more on teamwork, for complementary solutions. These additional skills usually increase a treatment's success and decrease complications, which, first and foremost, benefit the patients.

## 1 Introduction

Orthodontics is the branch of dentistry that deals with promoting correct craniofacial growth, the harmonious development of the jaws, and correct occlusion. It aims to eliminate any interference with these physiological processes or to correct dental misalignments, malocclusions, or skeletal discrepancies that may arise during growth or due to genetic and environmental factors (1–9). Orthodontic interventions play a fundamental role in ensuring the proper function and aesthetics of the stomatognathic system. The complexity of modern orthodontic care, which combines biological understanding, biomechanics, and digital technology, demands a high level of professional competence (10–32). Yet, despite its evolution, orthodontics is a relatively young specialty—barely over 100 years old (33–58) Advances such as 3D imaging, intraoral scanning, and computer-aided design (CAD) have dramatically transformed diagnostic and therapeutic workflows, enhancing precision and patient engagement (59–67).

Efforts to harmonize orthodontic education across Europe began with F. P. van der Linden's Erasmus project in 1992, recognizing the need for a common academic standard (68–78). Today, clinicians can pursue various pathways for advanced training, including residencies, university master's degrees, and continuing education courses. These educational opportunities are vital to ensure that practitioners stay up to date with emerging evidence, technologies, and treatment philosophies (79, 80).

In Italy, after the six-year degree in dentistry, a three-year graduate school of orthodontics is available to those who wish to specialize (81). This residency program provides structured, evidence-based training that includes theoretical instruction, clinical practice, and research. It enables young dentists to acquire the diagnostic skills and therapeutic competence needed to treat a wide variety of malocclusions. The program also emphasizes interdisciplinary collaboration, particularly with surgeons and prosthodontists, for complex cases (82–84).

In the absence of standardized training, dentists who follow non-residency routes may exhibit variable preparation and treatment outcomes, with potential repercussions for quality and patient safety (85).

Currently, there is limited literature addressing the specific role and importance of orthodontic residency in Italy and how such advanced training influences the clinical decisions of practitioners (86–94). Understanding whether formal postgraduate education significantly impacts the quality of care and treatment outcomes is essential. The purpose of this study is to explore this topic and help clarify how postgraduate orthodontic training shapes clinical practice and, ultimately, patient management (95–97).

## 2 Material and methods

A questionnaire was submitted to 500 Italian Dental offices, in which there was a possibility to receive an orthodontic treatment. Only 317 answers could be included in this study. The dentists were asked to complete an anonymous questionnaire (Table 1) electronically distributed, in Italian, between July and August 2022.

**Table 1:** Questionnaire.

Question	Possible answers
1. Do you have an orthodontic postgraduate degree?	Yes/No
2. Do you practice orthodontics exclusively?	Yes/No
3. How old are you?	
4. Do you have an Italian orthodontic postgraduate degree?	Yes/No
5. How many years ago did you receive your postgraduate degree?	
6. Do you have other postgraduate degrees?	Yes/No
7. Which type of orthodontic therapies did you use last year? (more than ten cases).	• Labial fixed appliances • Functional therapy • Aligners • Lingual fixed appliances • Elastodontic therapy
8. Which device did you mostly use last year for functional therapy? (more than ten cases).	• Rapid maxillar espander • Traditional expander • Twin block • Frankel • Delaire mask • Sander • Monoblock • Lip bumper • EGA (eruption guidance appliance) • High extraoral traction • Low extraoral traction • Herbst • Forsus
9. Which brackets did you mostly use, last year, for vestibular fixed orthodontic therapy? (more than ten cases) (you can choose more than one option).	• Roth brackets • MBT brackets • Roth self-ligating brackets • MBT self-ligating brackets • Damon self-ligating brackets • Rickets self-ligating brackets • Rickets brackets • Roncone technique • Alexander technique • Edgewise technique • Hilger System technique • Swing System technique • Tweed technique
10. In the last year you have used more: (more than ten cases).	• Straight wire • Segmented technique
11. Which kind of appliance did you mostly use last year for lingual orthodontic therapy? (more than ten cases).	• Incognito • Win • 2D
12. Which kind of aligners did you use most last year? (more than ten cases).	• Invisalign • Spark • Suresmile • OrthoCaps • ClearCorrect • Nuvola • Made in laboratory
13. Do you work alone or in a team when dealing with a diagnosis and treatment plan? (considering the last year).	• Team • Alone
14. Do you usually propose the aligners yourself or is the patient expressly requesting them? (considering the last year).	• I propose them • I wait for the patient to expressly request them
15. If you used the aligners last year, why did you do it? (you can choose more than one option).	• You consider the best choice from a clinical point of view and therefore of the result • You think this is the simplest therapy to set up • You think this is the quickest therapy • You think it is more beneficial to the clinician • You think it is more beneficial to the patient
15. Which type of patients did you mostly treat with aligners last year? (you can choose more than one option).	• Children • Teenagers • Adults

### 2.1 Dissemination

The Google Form link was distributed via email to dental offices across Italy and through professional contacts and mailing lists of dental associations. No reminders were sent.

### 2.2 Eligibility and data cleaning

Inclusion criteria were: licensed Italian dentist who practices orthodontics and complete questionnaire. Pre-specified internal validation and cross-item consistency checks were applied (e.g., implausible age vs. training timeline; contradictory answers). Eight questionnaires were excluded (5 implausible age declarations; 3 inconsistent answers), yielding 309 valid questionnaires for analysis.

### 2.3 Statistics

Categorical data are reported as frequencies/percentages and compared with Chi-square tests; effect size is Cramer's V. Numerical variables were compared with *t*-tests. Significance was set at *p* < 0.05.

### 2.4 Ethics and data protection

All respondents provided informed consent. Data were collected anonymously, in accordance with the Declaration of Helsinki.

## 3 Results

### 3.1 Sample and exclusions

After internal validation and consistency checks (see Methods), 309 questionnaires were retained for analysis (8 exclusions).

### 3.2 Specialization status

70% (218/312) were specialized in orthodontics and 30% (94/312) were not (Table 2).

**Table 2:** Italian orthodontic specialists.

Are you specialized in orthodontics?		Total, *n*
No, *n* (%)	Yes, *n* (%)	
94 (30%)	218 (70%)	312
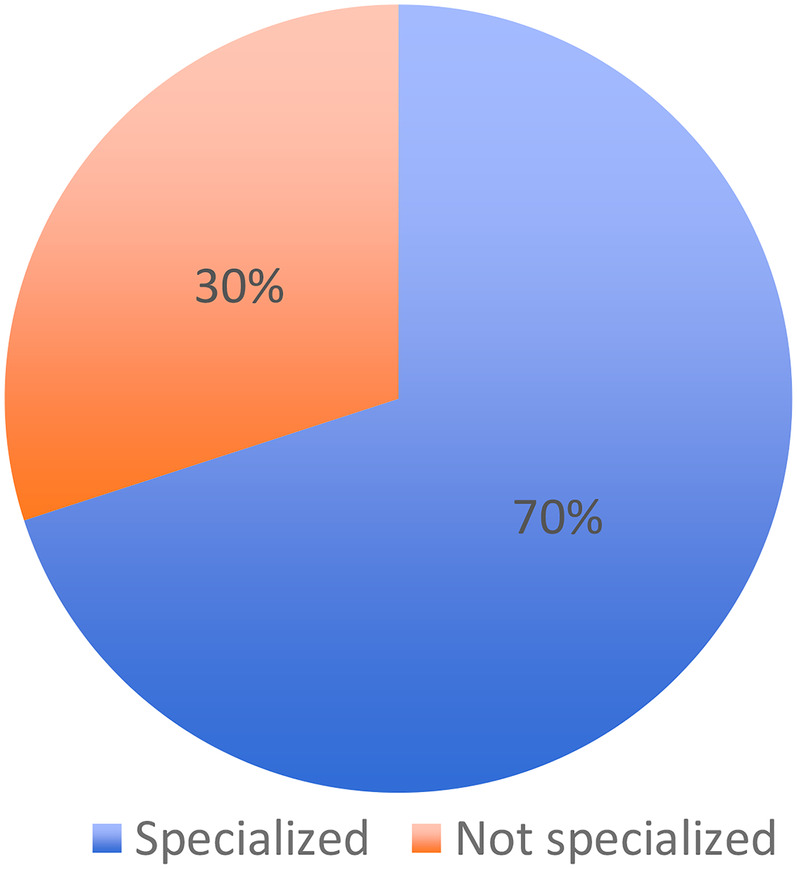		

### 3.3 Technique by specialization

Specialization was associated with technique choice (*χ*^2^ = 89.68, df = 2, *p* < 0.0001; Cramer's V = 0.54): specialists predominantly used straight wire, non-specialists mainly segmented tecnique (Table 3).

**Table 3:** Comparison of orthodontic techniques used by dentists with and without orthodontic specialization.

Do you specialize in orthodontics?	Technique used			Total, *n*
	Straight wire, n (%)	Both, *n* (%)	Segmented technique, *n* (%)	
No	28 (32%)	0 (0%)	60 (68%)	88
Yes	185 (84.8%)	3 (1,4%)	30 (13.8%)	218
Total	213 (69%)	3 (1%)	90 (30%)	306

### 3.4 Time since specialization

**Figure 1:**
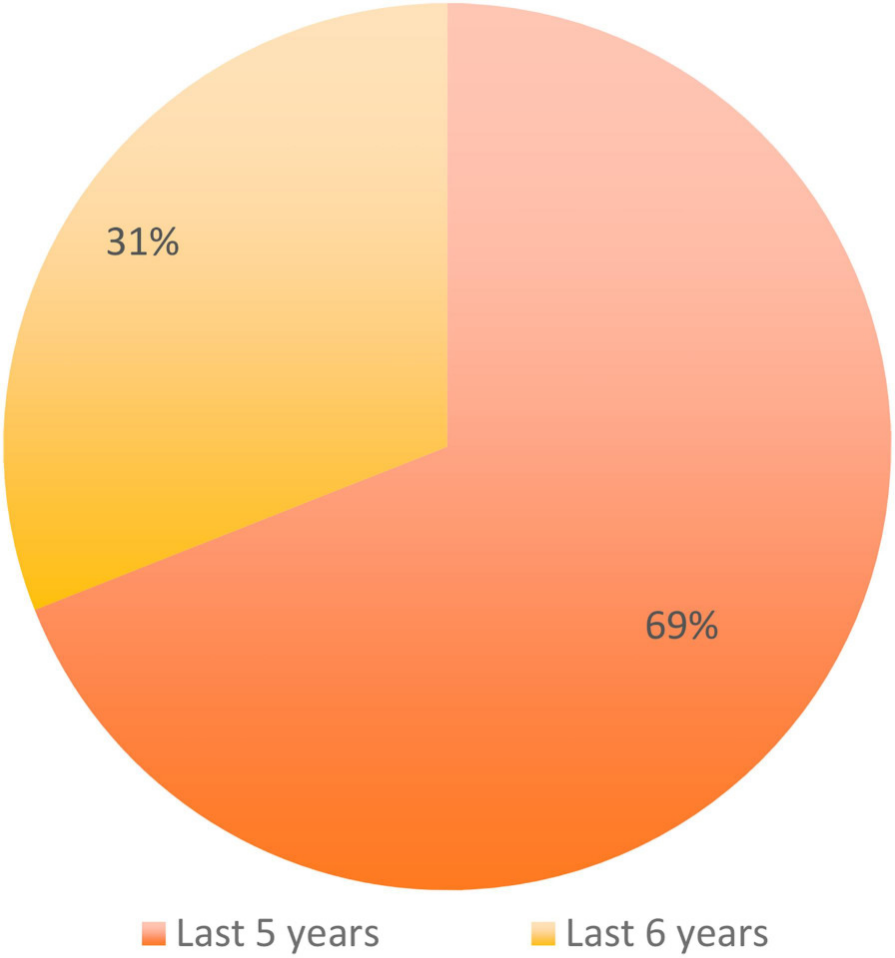
Distribution of respondents according to the time since obtaining orthodontic specialization (last 5 years vs more than 5 years).

Among specialists, 69% (158/229) obtained specialization ≤5 years ago and 31% (71/229) > 5 years (Table 4; Figure 1).

**Table 4:** Time since obtaining orthodontic specialization among respondents.

Specialized how long	Total
Last 5 Years	158 (69%)
More than 5 Years	71 (31%)
Total	229

### 3.5 Country of specialization

**Figure 2:**
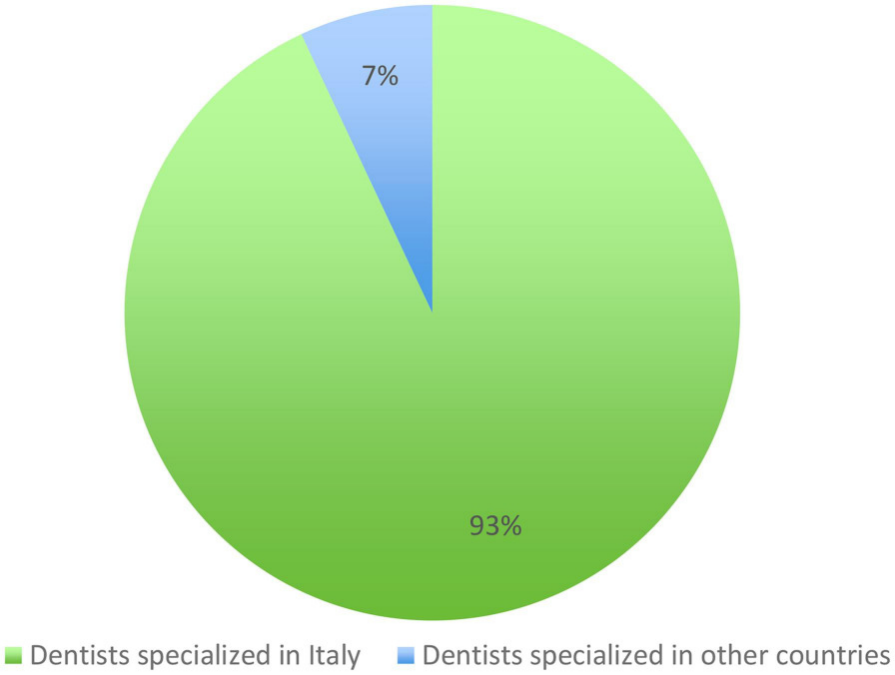
Distribution of respondents according to the country where orthodontic specialization was obtained (Italy vs abroad).

93% (203/218) trained in Italy and 7% (15/218) abroad (Table 5; Figure 2).

**Table 5:** Country where orthodontic specialization was obtained by respondents (Italy vs abroad).

Specialized in Italy	Total
No	15 (7%)
Yes	203 (93%)
Total	218

### 3.6 Lingual technique

Distribution of 2D, WIN, other/unknown did not differ by specialization (*p* = 0.96; Cramer's V≈0.03) (Table 6).

**Table 6:** Distribution of lingual orthodontic techniques (2D, WIN, other/unknown) used by specialists and non-specialists.

Do you specialize in orthodontics?	2D, *n* (%)	WIN, *n* (%)	Other/unknown, *n* (%)	Total, *n*
No	22 (29%)	26 (35%)	27 (36%)	75
Yes	52 (28%)	67 (36%)	65 (36%)	184
Total	74	93	92	259

### 3.7 Teamwork

Specialists were more likely to work in a team (77% vs. 23%); non-specialists were ∼even (51% vs. 49%); *χ*^2^ = 19.71, df = 1, *p* < 0.0001; Cramer's V≈0.26 (Table 7).

**Table 7:** Comparison between specialists and non-specialists in orthodontics regarding their preference for working alone or in a team.

Specialized in orthodontics?	Working in a team or alone		
	Alone	In a team	Total
No	44 (49%)	47 (51%)	91
yes	49 (23%)	170 (77%)	219
Total	93	217	310

### 3.8 Exclusive orthodontic practice

94% (206/218) of specialists reported only orthodontics, vs. 4% (4/93) of non-specialists (Table 8).

**Table 8:** Proportion of specialists and non-specialists in orthodontics who practice exclusively orthodontics.

Specialized in orthodontics?	Only orthodontics		
	No	Yes	Total
No	89 (96%)	4 (4%)	93
Yes	12 (6%)	206 (94%)	218
Total	101	210	311

### 3.9 Additional specializations

13% (28/217) of specialists reported an additional specialty (Table 9).

**Table 9:** Prevalence of additional specializations among orthodontic specialists.

More than 1 Specialty	Total
No	189 (87%)
Yes	28 (13%)
Total	217

### 3.10 Post-functional therapy choices

Among clinicians using functional therapy, specialists more often proceeded with fixed appliances (94% vs. 72%; Table 10) and used aligners slightly more often (64% vs. 60%; Table 11).

**Table 10:** Use of fixed orthodontic therapy after functional therapy among specialists and non-specialists.

Specialized in orthodontics?	Fixed therapy		
	No	Yes	Total
No	15 (28%)	38 (72%)	53
Yes	11 (6%)	174 (94%)	185
Total	26	212	238

**Table 11:** Use of aligners after functional therapy among specialists and non-specialists.

Specialized in orthodontics?	Aligners		
	No	Yes	Total
No	21 (40%)	32 (60%)	53
Yes	66 (36%)	119 (64%)	185
Total	87	151	238

### 3.11 Age

Mean age did not differ between predominant aligner users (44.4 years) and fixed-appliance users (45.15 years); *p* = 0.46.

Out of the whole sample, 242 clinicians use functional therapy (i.e., Frankel, Bionator, or Andresen), but while 133 of them, after functional therapy, apply both fixed orthodontic appliances (i.e., Straight wire, Tweed or Ricketts) and aligners, 79 use only fixed oral appliances, and 19 dentists use only an aligner. The kind of lingual technique used is independent from being a dentist with an orthodontic postgraduate degree or not. The data obtained suggests that specialists do not prefer a specific technique [V Cramer: 0.03; Chi Square Test (*p*-value) = 0.96]. The power of the Chi Square Test used is 95.1% for lingual techniques.

From the contingency table it is possible to note that, for those who don't have a specialty in orthodontics, there is an equal distribution between teamwork and work alone, while for the specialized ones there is a clear preference for teamwork. The Chi-Square Test leads us to accept the hypothesis of dependence between being specialized and preferring, or not, working with a team. The value of V Cramer equal to 0.258, however, indicates that there is no net dependence, in fact, as described above, those with specialization tend to work in a team while those without specialization do not have this attitude.

1. Among the respondents, 70% (218/312) were specialized in orthodontics, while 30% (94/312) were not (Table 2).
2. Which orthodontic techniques are the most used by those specialized and those not specialized in orthodontics? A significant association was found between specialization status and the orthodontic technique used (*χ*^2^ = 89.68, df = 2, *p* < 0.0001). Specialists predominantly used the straight wire technique, whereas non-specialists mainly adopted the segmented technique (Table 3).

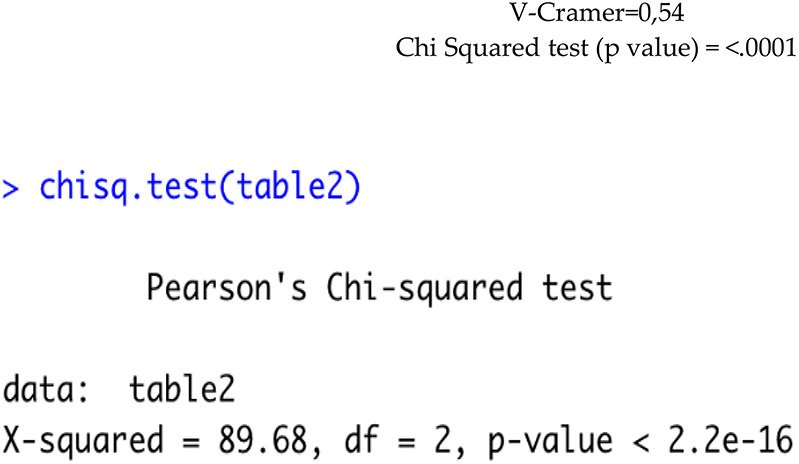


A Chi-squared test showed a significant association between specialization status and the orthodontic technique used (*χ*^2^ = 89.68, df = 2, *p* < 0.0001). We can observe that those who are specialized tend to use the straight wire technique more than those who are not specialized.

3. How many dentists have obtained a degree in orthodontics or other specialization in the last 5 years? (Table 4, Figure 1)
4. Who uses aligners the most and who uses traditional fixed orthodontics, based on age:

The average age of those who use aligners is 44.4 years, the average age of those who use fixed therapy is 45.15 years. Age difference is not significant (*t*-test for comparison of means, *p*-value = 0.46).

5. Do the dentists specializing in orthodontics prefer to use segmented technique or straight wire?

188 Dentists who specialized in orthodontics do prefer to use the straight wire orthodontic technique, while 33 prefer the use of segmented technique (3 of these prefer both techniques).

6. Did most dentists specializing in orthodontics choose their residency/degree in Italy or outside the country? (Table 5, Figure 2)
7. Which lingual technique is preferred by dentists with a degree in orthodontics? (Table 6)

The *p*-value is equal to 0.96, therefore no differences in the lingual technique are observed between those with and those with no specialization.

8. Comparison between the dentists with specialization and the ones without it, regarding the choice of working in a team or not (Table 7):

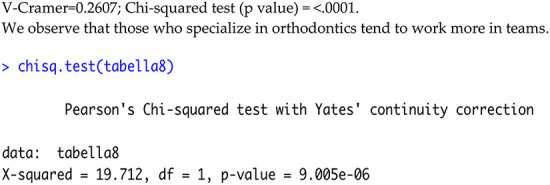


A Chi-squared test showed a significant association between specialization status and preference for working in a team (*χ*^2^ = 19.71, df = 1, *p* < 0.0001; Table 7). Specialists were more likely to work in teams compared to non-specialists.

9. Evaluate if those specialized in orthodontics practice only orthodontics (Table 8):

As we would expect, those who specialize in orthodontics are more inclined to work only in this field.

10. Evaluate if those who are specialized in orthodontics have or are pursuing a second specialization (Table 9):
11. Evaluate whether orthodontic specialists use functional orthodontics first and then aligners and/or fixed devices (Table 10):

*In the following tables, only the responses of those who have used functional therapy have been considered.

We observe that those who are specialized use fixed therapy much more than those who are not specialized (Table 11).

## 4 Discussion

Given the breadth of training options, orthodontists should emphasise prevention (98, 99). When treatment is required, a wide array of validated appliances can manage varied presentations (100–108).

Main findings. In this national survey, orthodontic specialization was strongly associated with technique selection and work organization: specialists predominantly used straight-wire mechanics and reported team-based care and exclusive orthodontic practice, whereas non-specialists more often adopted segmented tecnique and worked across broader scopes (109–111). No preference differences emerged for lingual systems.

### 4.1 Clinical implications of postgraduate education

Postgraduate training appears to shape clinicians' diagnostic repertoire, biomechanical planning, and practice organization. The higher use of straight-wire mechanics among specialists likely reflects greater confidence with comprehensive tooth movement and anchorage control acquired during residency, whereas the preference for segmented tecnique among non-specialists may represent a strategy to limit unwanted effects in complex movements when biomechanical mastery is more limited (112, 113). These patterns align with literature showing that outcomes and efficiency vary by appliance and operator proficiency, and that clear biomechanical planning remains decisive regardless of technique (114–119).

Specialization was also associated with team-based care. Interdisciplinary workflows—orthodontist with surgeons, pediatric dentists, myofunctional/speech therapists, etc.—are linked to safer, higher-quality care and more predictable management of complex cases (120–132). Finally, where aligners are used, evidence suggests advantages in oral hygiene and treatment comfort but mixed findings on movement accuracy in demanding biomechanics; successful aligner therapy depends on solid diagnostic and biomechanical competence—competencies typically emphasized in structured programs. Overall, our data supports the view that formal postgraduate education may translate into broader therapeutic options, greater integration with teams, and potentially more consistent treatment execution in daily practice (133).

### 4.2 Limitations of a self-reported cross-sectional survey

This study has important limitations. First, self-report introduces recall and social-desirability bias; responses were not verified against charts or objective outcomes. Second, although we applied internal validation and excluded inconsistent records, some misclassification is still possible and denominators vary across items. Third, the cross-sectional design precludes causal inference: specialization and technique choices may both be influenced by unmeasured factors (case mix, practice model, local market). Fourth, the sampling frame (email dissemination and professional networks) may entail selection bias and limits generalizability beyond Italy. Fifth, the questionnaire, while face-valid, lacked external psychometric validation (e.g., reliability indices). Finally, we did not collect clinical outcomes (e.g., ABO-OGS, treatment time, relapse, PROMs), so we cannot link training pathways to effectiveness or safety (134–136).

### 4.3 Suggestions for future research

Prospective and mixed-methods designs are needed to move beyond self-report and strengthen causal interpretation:

• Prospective cohorts/registries linking clinician training to objective outcomes (ABO-OGS, treatment duration, retreatment/relapse, root resorption, periodontal indices), with risk-adjustment for case complexity.
• Qualitative work (interviews/focus groups) to explore *why* some dentists do not pursue specialization and what drives technique selection (perceived risks/benefits, workload, economics, access to training).
• Comparative education studies across programs/countries (curricula, competencies, mentorship, clinical exposure) aligned with current European/WFO guidance.
• Instrument development: validation of a brief, reliable questionnaire (content/construct validity, test-retest) and clearer operational definitions for techniques.
• Health-services analyses: impact of team-based models on outcomes and costs; modeling of practice scope (exclusive vs. mixed) on adoption of innovations (e.g., aligners, TADs) (137–144).

Take-home messages. (i) Postgraduate education correlates with broader technique use and teamwork—organizational features linked to quality and safety (145–157). (ii) Self-reported, cross-sectional data cannot establish causality or outcomes. (iii) Future prospective and qualitative studies should test whether training pathways improve patient-level results and clarify barriers to specialization (158–165).

General dentists without orthodontic specialization tend to favour segmented technique for control of unwanted movements and may update techniques less often given their broader remit (166–173).

Our study has some limitations but also suggests opportunities for future research. With the exception of two potentially ambiguous items, the study did not elicit respondents’ reasons for not undertaking orthodontic specialization (174–180). Financial burden, universities not close to home, working in a rural area where the practitioner needs to have multiple but basic skills, or preference for general dental work may be some of the reasons. Conversely, behind the decision to get specialized could be the prospect of future higher earnings in orthodontics rather than in dentistry. Likewise, it's possible that financial burden on the patient and context in which an orthodontist works could impact the decision to use one orthodontic treatment over another (181–183).

## 5 Conclusions

Observing the data that emerged from this questionnaire submitted to 317 dentists/orthodontists, it is possible to conclude that pursuing a specialization in orthodontics brings advantages for both practitioners and patients: it gives orthodontists those extra skills to customize diagnosis and daily treatments in a more precise and innovative way, using a wider variety of therapeutic options and relying more on teamwork for complementary solutions. These additional skills usually increase treatment's success and decrease complications which, first and foremost, benefit the patients. A more specific diagnosis and a customized plan of care may reduce the time of treatment, avoid relapses, increase long term stability of the results and decrease the economic burden for the patient.

## Data Availability

The datasets presented in this study can be found in online repositories. The names of the repository/repositories and accession number(s) can be found in the article/Supplementary Material.
